# Validation of self‐reported influenza vaccination in the current and prior season

**DOI:** 10.1111/irv.12593

**Published:** 2018-08-14

**Authors:** Jennifer P. King, Huong Q. McLean, Edward A. Belongia

**Affiliations:** ^1^ Marshfield Clinic Research Institute Center for Clinical Epidemiology & Population Health Marshfield Wisconsin

**Keywords:** human, influenza, influenza vaccines, self‐report

## Abstract

Self‐reported influenza vaccination is generally accurate for the current season, but the accuracy of self‐report for vaccination in prior seasons is largely unknown. This study evaluated the accuracy of self‐report for current and prior season influenza vaccination among patients with medically attended acute respiratory illness enrolled in a study of influenza vaccine effectiveness during the 2014‐15 influenza season. It demonstrates there is a greater potential for exposure misclassification when prior season vaccinations are ascertained by self‐report. Percent agreement between self‐report and final status was high for both current and prior season vaccination: 97.7% and 93.2%, respectively.

## 1 BACKGROUND

Observational studies of influenza vaccine effectiveness (VE) are conducted annually in North America, Europe, and Australia. Accurate information on influenza vaccination status is important to minimize exposure misclassification and bias. These studies often rely on a combination of self‐report and information obtained from medical records to determine vaccination status. In the United States, influenza vaccines are readily available outside the healthcare system, and self‐report may be the only practical method to ascertain vaccination status in some research settings. This is especially true for ascertainment of vaccines received in prior seasons. Vaccine effectiveness may be reduced in persons who are repeatedly vaccinated, and recent VE studies have assessed this by collecting information on prior season vaccination. Self‐report of influenza vaccination is generally accurate for the current season, but the accuracy of self‐report for vaccination in prior seasons is largely unknown.^1^ Self‐report may be particularly susceptible to recall bias for more distant seasons and for individuals with sporadic vaccination patterns. We evaluated the accuracy of self‐reported influenza vaccination in the current season and the prior season among patients with medically attended acute respiratory illness who were enrolled in a study of influenza vaccine effectiveness during the 2014‐15 season.

## 2 METHODS

This study was conducted within an influenza vaccine effectiveness study in the 2014‐15 northern hemisphere influenza season. The methods of the influenza vaccine effectiveness study have been previously reported.^2^ In brief, patients with acute respiratory illness (including cough) with symptom duration ≤7 days were recruited during an outpatient visit. Enrollment was restricted to a predefined cohort of individuals living near Marshfield, Wisconsin, who receive care from Marshfield Clinic. This analysis is restricted to the individual's first enrollment in the season. After consent, adult participants and parents of children were interviewed to assess symptoms, onset date, and vaccination status. Participants were asked whether they had received a seasonal influenza vaccine since July 1, 2014. A separate question asked whether the previous season's influenza vaccine was received.

Vaccination status was ascertained from a regional immunization registry. The Registry for Effectively Communicating Immunization Needs (RECIN) is a web‐based, population‐based immunization registry to capture vaccines for both adults and children. RECIN is utilized by all public and private immunization providers serving the study population in central Wisconsin. The registry contains a longitudinal record of immunizations received by each individual, exchanges data weekly with the Wisconsin Immunization Registry, and employs record‐matching procedures to prevent duplicate entries. RECIN data include vaccine type, manufacturer, lot number, date of administration, and name/location of immunization provider. RECIN is integrated into the Marshfield Clinic electronic medical record and serves as the legal immunization record.

For this analysis, the accuracy of self‐reported vaccination was assessed relative to RECIN records at the conclusion of the season. If a RECIN record indicated receipt of influenza vaccine prior to enrollment in 2014‐15 or at any time during the 2013‐14 season, the patient was classified as vaccinated for the respective season. Records of patients who self‐reported no vaccination for which RECIN contained an influenza vaccination record were reviewed to confirm identity matching. If matching was confirmed, the RECIN record was accepted as valid. Participants who reported influenza vaccination in 2013‐14 or 2014‐15 without a corresponding RECIN record were interviewed by telephone. Those who re‐confirmed that they (or their child) received an influenza vaccine in the season(s) under investigation were asked to provide the date, location, and provider who administered the vaccine. Written documentation of vaccination was requested. Following consent for release of medical records, immunization providers were contacted to confirm vaccine receipt. For participants reporting receipt of influenza vaccine from Marshfield Clinic, health system electronic medical records were reviewed to confirm vaccination.

Participants were classified as vaccinated if (a) a record existed in RECIN or (b) written documentation of vaccine receipt was obtained from the immunization provider, or (c) health system electronic medical records confirmed vaccination. Participants were classified as unvaccinated if (a) there was no record of vaccine administration in RECIN and (b) confirmation of no vaccination was received from an immunization provider or health system electronic medical records had no evidence of vaccination.

We calculated percent agreement and kappa between self‐reported and adjudicated vaccination status. The sensitivity was defined as the percent of vaccinated individuals correctly identified as vaccinated by self‐report. Specificity was defined as the percent of unvaccinated individuals correctly identified as unvaccinated by self‐report. Participants were excluded if responding “don't know” to vaccination status, refusing participation, unable to be reached, unable to provide sufficient detail for contacting the immunization provider, or if, after 3 attempts, authorization releases or verification documents were not returned. Children under 6 months of age as of September 1, 2013 were excluded from the prior season analysis as they were not eligible for vaccination. All analyses were performed with SAS 9.4.

The study was approved by the Marshfield Clinic Institutional Review Board, and informed consent was obtained from all participants.

## 3 RESULTS

**Figure 1:**
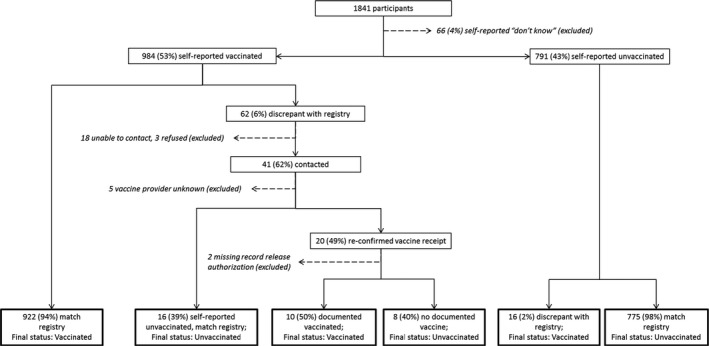
Adjudication of current season influenza vaccination status, 2014‐15

The analysis included 1841 patients with acute respiratory illness who were enrolled in the 2014‐15 vaccine effectiveness study; 1683 (91%) were non‐Hispanic white, 89 (5%) were Hispanic, 1025 (56%) were female, and 994 (54%) were aged <18 years. Nine hundred eighty‐four participants (53%) initially reported receiving the 2014‐15 influenza vaccine, and 922 (94%) of these were confirmed by a vaccination record RECIN. There were 62 participants who reported vaccine receipt without a corresponding record in RECIN (Figure 1). Among 791 participants who denied receipt of the 2014‐15 vaccine, 2% had a vaccine record in RECIN. Vaccination dates for those denying vaccination spanned the entire season, and their enrollment dates ranged from December 2014 to March 2015.

We contacted 41 of 62 participants who self‐reported vaccination in the 2014‐15 season without RECIN confirmation. Twenty re‐confirmed vaccine receipt and provided authorization to request records. Sixteen individuals stated that their initial self‐report of vaccination was incorrect, and they had not received the 2014‐15 vaccine. Following adjudication, 10 participants were reclassified as vaccinated based on requested documentation. Overall agreement between self‐report and final status was 97.7% (Kappa 0.95) (Table 1). The sensitivity of self‐report for current season vaccine receipt was 98.3% overall. It was lowest in children 5‐17 years old (97.0%) and highest (100%) in adults 18‐49 years old. Specificity was 97.0% overall, highest in adults 50‐64 years of age (98.8%), and lowest in children under 5 years old (94.6%).

**Table 1:** Self‐reported current season influenza vaccination status as compared to adjudicated vaccination status, 2014‐15

Current season								
Self‐report	Adjudicated vaccination status		Percent Agreement	Kappa	Sensitivity (95% CI)	Specificity (95% CI)	PPV (95% CI)	NPV (95% CI)
	Vaccinated	Unvaccinated						
Age 6‐59 mo								
Vaccinated	239	8	96.2%	0.92	97.2%	94.6%	96.8%	95.2%
Unvaccinated	7	140			(94.2%‐98.9%)	(89.6%‐97.6%)	(93.7%‐98.6%)	(90.4%‐98.1%)
Age 5‐17 y								
Vaccinated	225	10	96.8%	0.94	97.0%	96.7%	95.7%	97.7%
Unvaccinated	7	293			(93.9%‐98.8%)	(94.0%‐98.4%)	(92.3%‐97.9%)	(95.3%‐99.1%)
Age 18‐49 y								
Vaccinated	170	4	99.0%	0.98	100%	98.2%	97.7%	100%
Unvaccinated	0	220			(97.9%‐100%)	(95.5%‐99.5%)	(94.2%‐99.4%)	(98.3%‐100%)
Age 50‐64 y								
Vaccinated	141	1	99.1%	0.98	99.3%	98.8%	99.3%	98.8%
Unvaccinated	1	81			(96.1%‐100%)	(93.3%‐100%)	(96.1%‐100%)	(93.4%‐100%)
Age 65 + y								
Vaccinated	157	1	99.0%	0.97	99.4%	97.6%	99.4%	97.6%
Unvaccinated	1	41			(96.5‐100%)	(87.4%‐99.9%)	(96.5%‐100%)	(87.4%‐100%)
All ages								
Vaccinated	932	24	97.7%	0.95	98.3%	97.0%	97.5%	98.0%
Unvaccinated	16	775			(97.3%‐99.0%)	(95.6%‐98.1%)	(96.3%‐98.4%)	(96.7%‐98.8%)

**Figure 2:**
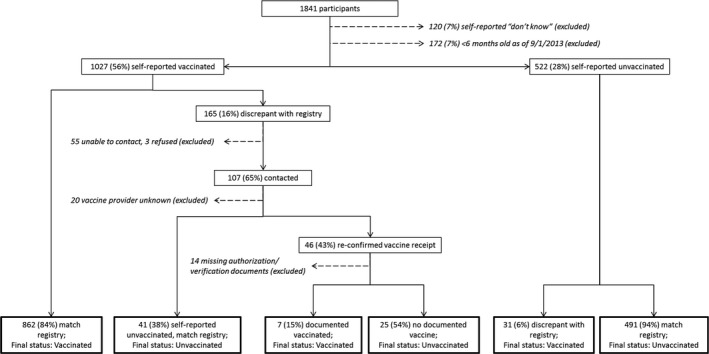
Adjudication of prior season (2013‐14) influenza vaccination status at the conclusion of the 2014‐15 season

For the prior (2013‐14) season (Figure 2), 165 participants (16%) reported prior season vaccination without a corresponding record in RECIN. Of 522 participants who denied receipt of 2013‐14 vaccine, 6% had a vaccine record in RECIN. The recorded vaccination dates were from across the 2013‐14 season and enrollments spanned the entire 2014‐15 season.

We contacted 107 of 165 participants self‐reporting vaccination in the 2013‐14 season without RECIN confirmation. Forty‐six re‐confirmed vaccine receipt and provided authorization to request records. Forty‐one individuals stated that their initial self‐report of vaccination was incorrect, and they had not received the 2013‐14 vaccine. Following adjudication, 7 participants were reclassified as vaccinated based on requested documentation. Overall agreement between self‐report and final status was 93.3% (Kappa 0.86) (Table 2). The sensitivity of self‐report for prior season vaccine receipt was 96.6% overall. It was lowest (93.2%) in children 6‐59 months old and highest (98.7%) in adults ≥65 years old. Specificity was 88.2% overall, highest (92.4%) in adults 50‐64 years of age, and lowest (86.6%) in children 5‐17 years old.

**Table 2:** Self‐reported previous season (2013‐14) influenza vaccination status as compared to adjudicated previous season vaccination status, 2014‐15

Past season								
Self‐report	Adjudicated vaccination status		Percent agreement	Kappa	Sensitivity (95% CI)	Specificity (95% CI)	PPV (95% CI)	NPV (95% CI)
	Vaccinated	Unvaccinated						
Age 6‐59 mo								
Vaccinated	150	6	92.2%	0.80	93.2%	89.5%	96.2%	82.3%
Unvaccinated	11	51			(88.1%‐96.5%)	(78.5%‐96.0%)	(91.8%‐98.6%)	(70.5%‐90.8%)
Age 5‐17 y								
Vaccinated	247	29	92.8%	0.85	98.0%	86.6%	89.5%	97.4%
Unvaccinated	5	188			(95.4%‐99.4%)	(81.4%‐90.9%)	(85.3%‐92.9%)	(94.1%‐99.2%)
Age 18‐49 y								
Vaccinated	177	22	91.5%	0.83	95.2%	87.6%	88.9%	94.5%
Unvaccinated	9	155			(91.0%‐97.8%)	(81.8%‐92.0%)	(83.7%‐92.9%)	(89.8%‐97.5%)
Age 50‐64 y								
Vaccinated	139	5	95.7%	0.90	97.2%	92.4%	96.5%	93.9%
Unvaccinated	4	61			(93.0%‐99.2%)	(83.2%‐97.5%)	(92.1%‐98.9%)	(85.0%‐98.3%)
Age 65 + y								
Vaccinated	156	4	97.0%	0.94	98.7%	90.0%	97.5%	94.7%
Unvaccinated	2	36			(95.5%‐99.9%)	(76.3%‐97.2%)	(93.7%‐99.3%)	(82.3%‐99.4%)
All ages								
Vaccinated	869	66	93.3%	0.86	96.6%	88.2%	92.9%	94.1%
Unvaccinated	31	491			(95.2%‐97.7%)	(85.2%‐90.7%)	(91.1%‐94.5%)	(91.7%‐95.9%)

## 4 DISCUSSION

This study confirms previous, published reports indicating that self‐report of influenza vaccination in the current season can provide a valid measure of vaccine exposure when medical records or registry data are not available.^1^, ^3^, ^4^ To our knowledge, this is the first study to assess the accuracy of self‐reported influenza vaccination in the prior season. We observed a higher level of misclassification for self‐reported vaccination in the prior season, although the percent agreement was above 90% for all age groups. The sensitivity of self‐reported vaccination in the prior season was also greater than 90% in all age groups. The specificity of self‐report (ie, 100 × self‐reported unvaccinated/all unvaccinated) was substantially lower for the prior season (88.2%) compared to the current season (97.0%). These findings demonstrate a greater potential for bias in analyses that rely on self‐report of vaccination in the prior season.

The lowest percent agreement for report of prior season vaccination status was seen in 18‐ to 49‐year‐olds. Misclassification occurred in nearly 10% of reports in this population. However, for the current season, the percent agreement for the same age group was among the highest, with no one incorrectly reported as unvaccinated and only 2% incorrectly reported as vaccinated without substantiation. This age group had the lowest influenza vaccination coverage in the United States in both 2013‐14 and 2014‐15 and was the last age group recommended for annual influenza vaccination.^5^, ^6^ This population is also likely to have sporadic vaccination patterns making recall of prior season vaccination more subject to error.

Adults aged 50 years and over had the highest agreement in both the current and prior season. These findings are concordant with studies showing high reliability of and high sensitivities for self‐report of influenza vaccinations in older adults.^4^, ^7^ Older adults were among the first groups recommended for annual vaccination and often have more frequent contact with the healthcare system so may have more consistent annual vaccination which facilitates recall.

In both seasons, the number of vaccinated individuals denying vaccination was less than those reporting vaccination without documentation. There was no observable pattern of late‐season enrollments coupled with early season vaccination which might suggest recall bias within a season, but this misclassification did occur more for prior season vaccination than current season.

A limitation of this study is the lack of racial/ethnic diversity. Results may not be generalizable to more diverse urban populations. We were also unable to obtain responses from all vaccination providers, particularly companies hired by employers to implement workplace vaccination campaigns, which led to exclusions from the analyses.

In conclusion, this study demonstrates a greater potential for exposure misclassification when prior season vaccinations are ascertained by self‐report. The impact of this exposure misclassification on vaccine effectiveness estimates requires further assessment.

## References

[1] Irving SA , Donahue JG , Shay DK , Ellis‐Coyle TL , Belongia EA . Evaluation of self‐reported and registry‐based influenza vaccination status in a Wisconsin cohort. Vaccine. 2009;27(47):6546‐6549. 19729083 10.1016/j.vaccine.2009.08.050

[2] McLean HQ , Thompson MG , Sundaram ME , et al. Influenza vaccine effectiveness in the United States during 2012‐2013: variable protection by age and virus type. J Infect Dis. 2015;211(10):1529‐1540. 25406334 10.1093/infdis/jiu647 PMC4407759

[3] Poehling KA , Vannoy L , Light LS , et al. Assessment of parental report for 2009‐2010 seasonal and monovalent H1N1 influenza vaccines among children in the emergency department or hospital. Acad Pediatr. 2012;12(1):36‐42. 22033102 10.1016/j.acap.2011.08.006 PMC3261370

[4] Laurence A , Lewis P , Gately C , Dixon A . Influenza and pneumococcal vaccination: do older people know if they have been vaccinated? Aust N Z J Public Health. 2016;40(3):279‐280. 26261068 10.1111/1753-6405.12423

[5] CDC . Flu Vaccination Coverage, United States, 2013‐14 Influenza Season. 2014 https://www.cdc.gov/flu/fluvaxview/coverage-1314estimates.htm. Accessed April 16, 2018.

[6] CDC . Flu Vaccination Coverage, United States, 2014‐15 Influenza Season. 2015 https://www.cdc.gov/flu/fluvaxview/coverage-1415estimates.htm. Accessed April 16, 2018.

[7] Mac Donald R , Baken L , Nelson A , Nichol KL . Validation of self‐report of influenza and pneumococcal vaccination status in elderly outpatients. Am J Prev Med. 1999;16(3):173‐177. 10198654 10.1016/s0749-3797(98)00159-7

